# Supplementary material for: The Systems Biology Research Tool: evolvable open-source software
Source: BMC Syst Biol. 2008 Jun 29;2:55. doi: 10.1186/1752-0509-2-55 (PMC2446383; doi:10.1186/1752-0509-2-55)
Supplement: Additional file 1 — SBRT Archive. An archive of the current version of the Systems Biology Research Tool. [file 1752-0509-2-55-S1.zip › sbrt-1.4.0/doc/developers_guide/api/sbrt/shell/io/DevNull.html]

DevNull


|  |  |  |
| --- | --- | --- |
| **Overview** \| **Package** \| **Class** \| **Use** \| **Tree** \| **Deprecated** \| **Index** \| **Help** | |  |
| **PREV CLASS**   **NEXT CLASS** | **FRAMES**    **NO FRAMES**     **All Classes** |  |
| SUMMARY: NESTED \| FIELD \| CONSTR \| METHOD | DETAIL: FIELD \| CONSTR \| METHOD |  |


---


## sbrt.shell.io Class DevNull

```
java.lang.Object
  java.io.OutputStream
      java.io.FilterOutputStream
          java.io.PrintStream
              sbrt.shell.io.DevNull
```

**All Implemented Interfaces:**: java.io.Closeable, java.io.Flushable, java.lang.Appendable

---

``` public class DevNull extends java.io.PrintStream ```

This class is used to mimic /dev/null from Unix-like
operating systems. An instance of
this class discards all data written to it.

**Author:**
:   This class was written and documented by
    Jeremiah Wright while in the Wagner lab.

---

**Field Summary**

**Fields inherited from class java.io.FilterOutputStream**

`out`


**Constructor Summary**

`DevNull()`
  
          Constructs new "/dev/null" object.


**Method Summary**

**Methods inherited from class java.io.PrintStream**

`append, append, append, checkError, close, flush, format, format, print, print, print, print, print, print, print, print, print, printf, printf, println, println, println, println, println, println, println, println, println, println, setError, write, write`

**Methods inherited from class java.io.FilterOutputStream**

`write`

**Methods inherited from class java.lang.Object**

`clone, equals, finalize, getClass, hashCode, notify, notifyAll, toString, wait, wait, wait`

**Constructor Detail**

### DevNull

```
public DevNull()
```

:   Constructs new "/dev/null" object.


---


|  |  |  |
| --- | --- | --- |
| **Overview** \| **Package** \| **Class** \| **Use** \| **Tree** \| **Deprecated** \| **Index** \| **Help** | |  |
| **PREV CLASS**   **NEXT CLASS** | **FRAMES**    **NO FRAMES**     **All Classes** |  |
| SUMMARY: NESTED \| FIELD \| CONSTR \| METHOD | DETAIL: FIELD \| CONSTR \| METHOD |  |


---
